# Supplementary material for: High-Mobility Free-Standing InSb Nanoflags Grown on InP Nanowire Stems for Quantum Devices
Source: ACS Appl Nano Mater. 2021 May 26;4(6):5825–33. doi: 10.1021/acsanm.1c00734 (PMC8291043; doi:10.1021/acsanm.1c00734)
Supplement: Supplementary file 1 — an1c00734_si_001.pdf [file an1c00734_si_001.pdf]

Supporting Information

"High Mobility Free-Standing InSb Nanoflags Grown on InP Nanowire Stems for Quantum Devices"

Isha Verma[1], Sedighe Salimian[1], Valentina Zannier[1], Stefan Heun[1], Francesca Rossi[2], Daniele Ercolani[1*], Fabio Beltram[1], and Lucia Sorba[1]*

[1] NEST, Istituto Nanoscienze-CNR and Scuola Normale Superiore, Piazza San Silvestro 12, I-56127 Pisa, Italy

[2] IMEM-CNR, Parco Area delle Scienze 37/A, I-43124 Parma, Italy

Contents

S1. InSb NFs on InAs stem vs InP stem

S2. EDX and HRTEM analysis of the InP-InSb NF interface

S3. Four-probe field effect mobility

S4. Hall mobility

[1]*correspondence email: valentina.zannier@nano.cnr.it, daniele.ercolani@sns.it

S1. InSb NFs on InAs stem vs InP stem

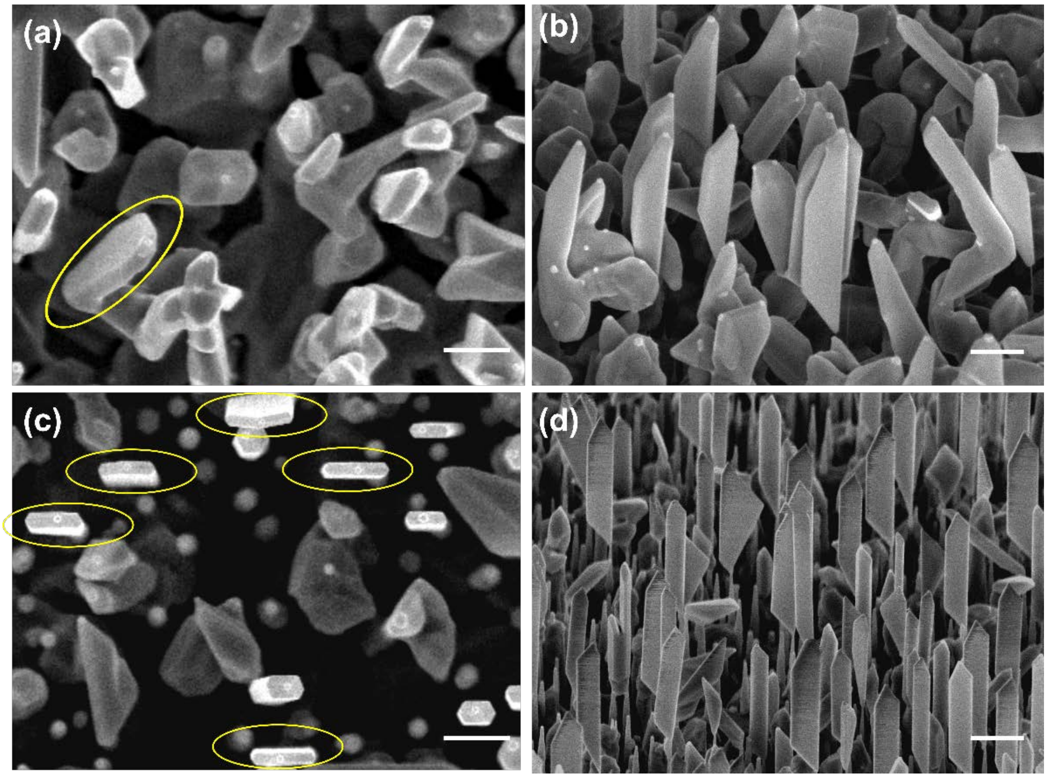

Figure S1. InSb NFs on InAs vs InP stem. Top view SEM images of InSb NFs on (a) InAs and (c) InP NW stems. 45°-tilted view SEM images of InSb NFs on (b) InAs stems and (d) InP NW stems. Scale bar is 500 nm in all panels. The InSb NFs that have (w/t) ≥ 4 are marked by yellow circles.

Figure S1 shows as-grown InSb NFs on InAs stems (top view and 45°-tilted view SEM image in panel (a) and (b), respectively), while on InP NW stems in panel (c) and (d). The growth protocol is the same as illustrated in panel (a) of Fig. 4 in the main text. The criterium for selection of preferred InSb NFs for fabricating quantum devices is defined by the following parameter:

$$\frac{Width\ of\ InSb\ NFs}{Thickness\ of\ InSb\ NFs} = \frac{w}{t} \geq 4$$

Counting the NFs satisfying this condition (marked with yellow circles in figure S1), we get a yield of 1% for InSb NFs grown on InAs stems, while for those grown on InP stems, the yield is 40%. This demonstrate a clear advantage for using robust tapered InP NW stems instead of

thin untapered InAs stems. Indeed, for long InSb growth times, the thin untapered InAs stems bend, leading to the loss of alignment with the precursor fluxes and consequently of the InSb orientation. Therefore, the preferential growth direction vanishes, and more 3D-like InSb structures are obtained.

S2. EDX and HRTEM analysis of the InP-InSb NF interface

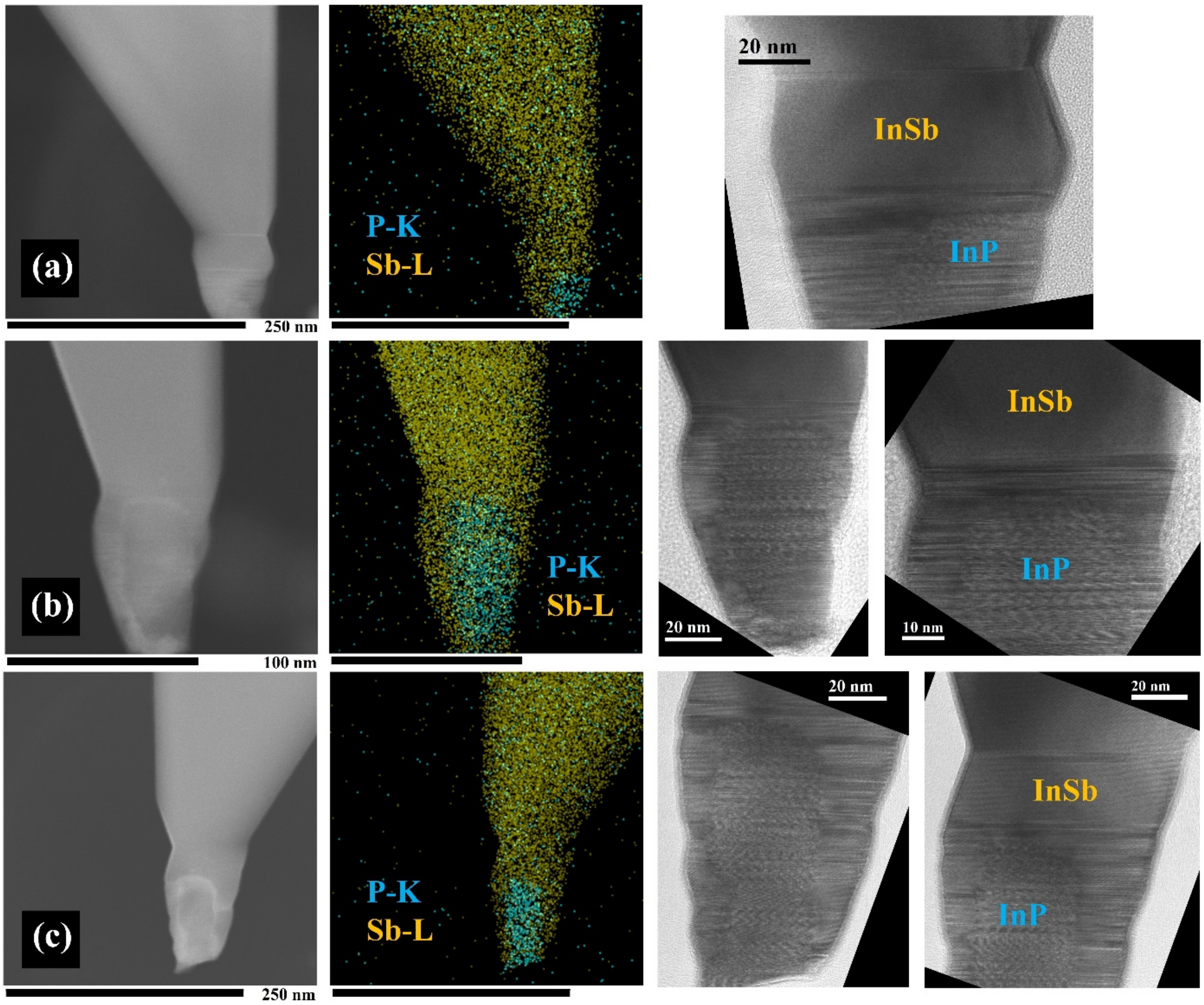

Figure S2. (a-c) EDX and HRTEM analysis of the basal region of three representative nanoflags, showing both axial and radial growth of InSb on the InP NW stem. From left to right: STEM-HAADF image, corresponding EDX map of the Sb (dark yellow) and P (blue) distributions, and HRTEM images of the interface.

Figure S2 shows STEM-EDX and HRTEM images of the basal region of three representative NFs, showing both axial and radial growth of InSb on the InP NW stem. Since the as-grown InP NW stem has a mixed wurtzite/zinc blende structure, stacking defects are clearly visible. The InSb radial shell is grown in either an asymmetric or a symmetric fashion around the InP stem (see panels (a) to (c)) and it retains the stacking defects of the InP NW stem (clearly visible from the HRTEM images). This radial InSb shell makes the top part of the InP NW stem thicker, adding further support for the growth of large InSb NFs.

S3. Four-probe field effect mobility

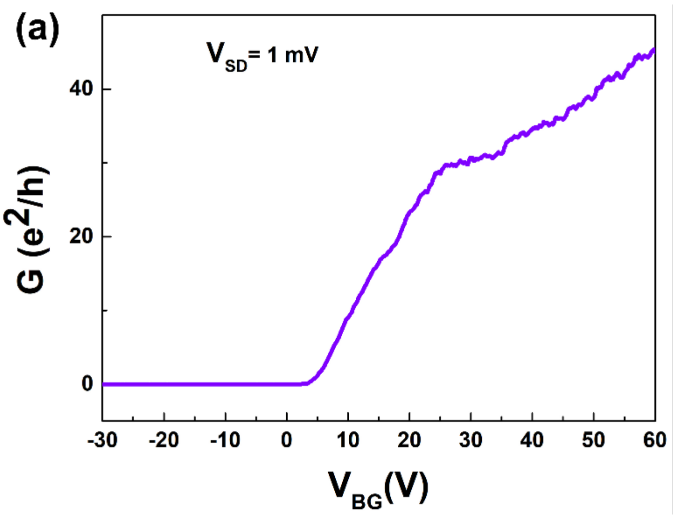

Figure S3. (a) Conductance G versus back gate voltage. The measurement was performed at a temperature of 4.2 K.

In a four-probe measurement under constant AC voltage bias at 4.2 K, the variation of the injected current is measured as shown in Figure 6(c) of the main text, and the longitudinal voltage drop V_{xx} (using contacts 1-2) is measured, as well. This allows to calculate the conductance $G = I_{SD}/V_{xx}$ as shown in Figure S3(a). The four-probe field effect mobility is then obtained using the formula

$$\mu_{4p\ FE} = \frac{L}{WC_{ox}}\left(\frac{dG}{dV_{BG}}\right), \qquad \text{(S1)}$$

with L/W = 4.6 and C_{ox} = 10 nF/cm^2. The four-probe field effect mobility obtained is 28000 cm^2/Vs. The n-type trend of the four-probe charge carrier modulation is in agreement with Fig. 6(c), since the conductivity increases with increasingly positive back gate voltages.

S4. Hall mobility

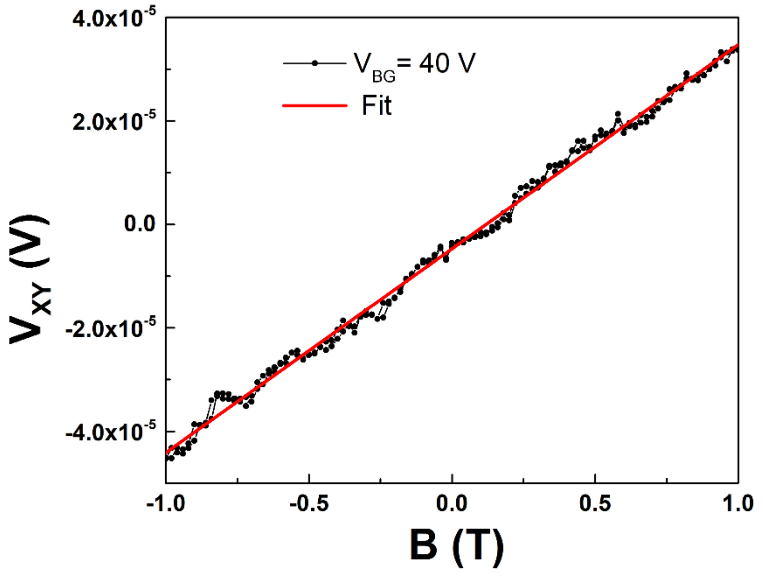

Figure S4. The transversal voltage drop V_{xy} as a function of magnetic field B at V_{BG} = 40 V and T = 4.2 K.

We measured the Hall voltages as a function of magnetic field ranging from -1T to +1T under constant AC current bias of 100 nA for different back gate voltages. We measured the Hall-bar device shown in Figure 6(a), with the channel width (width between contacts 1-3 and 2-4) of 325 nm and the channel length (1-2 and 3-4) of 1.5 μm. The naoflag thickness is ~100 nm. An example in Figure S4 shows V_{xy} for V_{BG} = 40 V. The figure shows the forward and backward sweep of the magnetic field, to demonstrate the reproducibility of the measurement, plus the fit to the experimental data, from which the carrier concentration is obtained. In detail, carrier concentration n and Hall mobility μ_H for each back gate voltage are calculated using the formulas

$$\mu_H = \frac{L}{W\langle V_{xx}\rangle}\left(\frac{V_{xy}}{B}\right) \qquad \text{(S2)}$$

$$n = \frac{I}{e}\left(\frac{B}{V_{xy}}\right) \qquad \text{(S3)}$$

with e the elementary charge.
